# Japanese translation and linguistic validation of the US National Cancer Institute’s Patient-Reported Outcomes version of the Common Terminology Criteria for Adverse Events (PRO-CTCAE)

**DOI:** 10.1186/s41687-017-0012-7

**Published:** 2017-12-05

**Authors:** Tempei Miyaji, Yukiko Iioka, Yujiro Kuroda, Daigo Yamamoto, Satoru Iwase, Yasushi Goto, Masahiro Tsuboi, Hiroki Odagiri, Yu Tsubota, Takashi Kawaguchi, Naoko Sakata, Ethan Basch, Takuhiro Yamaguchi

**Affiliations:** 1 Department of Clinical Trial Data Management, Graduate School of Medicine, The University of Tokyo, 7-3-1 Hongo, Bunkyo-ku, Tokyo, 113-8655 Japan; 2 Division of Health Care Research, QOL Research Group, Center for Public Health Sciences, National Cancer Center, 5-1-1 Tsukiji, Chuo-ku, Tokyo, 104-0045 Japan; 3 Adult Nursing, Chronic Illness and Conditions Nursing, St. Luke’s College of Nursing, 10-1 Akashi-cho, Chuo-ku, Tokyo, 104-0044 Japan; 4 Graduate School of Health and Social Services, Saitama Prefectural University, 820 San-Nomiya, Koshigaya-shi, Saitama, 343-8540 Japan; 5 Department of Public Health, Fukushima Medical University, 1 Hikariga-oka, Fukushima City, Fukushima 960-1295 Japan; 6 Breast unit, Kansai Medical University Medical Center, 10-15 Fumizono-cho, Moriguchi-city, Osaka 570-8507 Japan; 7 Department of Palliative Medicine, The Institute of Medical Science, The University of Tokyo, 4-6-1 Shirokanedai, Minato-ku, Tokyo, 108-8639 Japan; 8 Department of Thoracic Oncology, National Cancer Center Hospital, 5-1-1 Tsukiji, Chuo-ku, Tokyo, 104-0045 Japan; 9 Division of Thoracic Surgery, National Cancer Center East Hospital, 6-5-1 Kashiwanoha, Kashiwa, Chiba 277-8577 Japan; 10 Division of Breast Surgery, Hirosaki National Hospital, 1 Tomino-cho, Hirosaki, Aomori, 036-8545 Japan; 11 Department of Surgery, Kansai Medical University, 10-15 Fumizonochō, Moriguchi-shi, Osaka, 570-0074 Japan; 12 Department of Practical Pharmacy, School of Pharmacy, Tokyo University of Pharmacy and Life Sciences, 1432-1, Horinouchi, Hachioji-city, Tokyo, 192-0392 Japan; 13 Department of Palliative Medicine, The University of Tokyo Hospital, 7-3-1 Hongo, Bunkyo-ku, Tokyo, 113-8655 Japan; 14 Department of Medicine, University of North Carolina at Chapel Hill, 321 S Columbia St, Chapel Hill, NC 27516 USA; 15 Division of Biostatistics, Tohoku University Graduate School of Medicine, 1-1 Seiryo-machi, Aoba-ku, Sendai, Miyagi 980-8575 Japan

**Keywords:** PRO-CTCAE, Japanese translation, Linguistic validation, Adverse events, Patient-reported outcomes

## Abstract

**Background:**

The US National Cancer Institute (NCI) has developed the Patient-Reported Outcomes version of the Common Terminology Criteria for Adverse Events (PRO-CTCAE) to capture patients’ self-reported symptomatic adverse events in cancer clinical trials. The aim of this study was to develop and linguistically validate a Japanese translation of PRO-CTCAE. Forward- and back-translations were produced, and an independent review was performed by the Japan Clinical Oncology Group (JCOG) Executive Committee and the US NCI. We then conducted cognitive interviews with 21 patients undergoing cancer treatment. Participants were asked to complete the PRO-CTCAE and were interviewed using semi-structured scripts and predetermined probes to investigate whether any items were difficult to understand or answer. The interviews were recorded and transcribed, and a thematic analysis was performed. The data were split into two categories: 1) remarks on the items and 2) remarks on the questionnaire in general.

**Results:**

Twenty-one cancer patients undergoing chemotherapy or hormone therapy were interviewed at the University of Tokyo Hospital and the Kansai Medical University Hirakata Hospital during 2011 and 2012. Thirty-three PRO-CTCAE items were evaluated as “difficult to understand,” and 65 items were evaluated as “difficult to answer” by at least one respondent. However, on further investigation, only 24 remarks were categorized as “comprehension difficulties” or “clarity” issues. Most of these remarks concerned patients’ difficulties with rating their experience of individual symptomatic events.

**Conclusions:**

The study provides preliminary evidence supporting the linguistic validity of the Japanese version of PRO-CTCAE. Further cognitive interviewing is warranted for PRO-CTCAE items relating to sexuality and anxiety and for response options on severity attribute items.

## Background

The US National Cancer Institute (NCI) Common Toxicity Criteria for Adverse Events (CTCAE) is a longstanding grading system widely used for evaluating an array of adverse events in cancer treatment. There is a growing awareness of the importance of patients’ self-reporting of symptoms using patient-reported outcome (PRO) tools in addition to professional assessments [1–3]. In order to improve the accuracy and efficiency of collecting and grading adverse event data, the NCI has established the Patient-Reported Outcomes version of the Common Terminology Criteria for Adverse Events (PRO-CTCAE) measurement system [4–7]. The PRO-CTCAE item library contains 124 items reflecting 78 symptomatic adverse events drawn from the CTCAE version 4.0. The PRO-CTCAE was developed to be used in conjunction with the CTCAE [7]. PRO-CTCAE symptom attributes include frequency (F) (e.g., “In the last 7 days, how often did you have nausea?”), severity (S) (e.g., “In the last 7 days, what was the severity of your pain?”), interference with daily activities (I) (e.g., “How much did fatigue interfere with your usual or daily activities?”), and presence/absence/amount (P) (e.g., “In the last 7 days, did you have any bed sores?”*)*. PRO-CTCAE scores range from 0 to 4, corresponding to the response choices. The recall period for each question is the previous 7 days [8]. Content validation and a psychometric evaluation of the PRO-CTCAE measurement system have already been performed [6, 9] and their feasibility investigated in a multicenter oncology trial [10]. In addition, cognitive interview-based validation was performed in an adolescents-with-cancer population [11] and a pediatric and prosy version of the PRO-CTCAE is also being developed [12]. As part of the development of PRO-CTCAE, the NCI has collaborated on the development of versions of PRO-CTCAE in multiple other languages, such as Spanish [13], German [14], and Danish [15] language versions. A Japanese-language version was also created, in a joint endeavor by Tohoku University, The University of Tokyo, and the Japan Clinical Oncology Group (JCOG). The purpose of the Japanese translation is to encourage the use of PRO-CTCAE among Japanese-speaking cancer patients living in the USA, as well as those in Japan [16].

Estimated cancer incidence in Japan in 2016 was approximately one million, and estimated cancer deaths were 374,000. It is estimated that one in two Japanese will be diagnosed with cancer during their lifetime [17]. Accessing subjective symptoms of Japanese cancer patients by self-report has come to be viewed as important as the objective clinical endpoints such as response rate, disease-free survival, and overall survival [18]. The aim of this study was to translate the PRO-CTCAE into Japanese and linguistically validate the Japanese version.

### Linguistic validation

The term “linguistic validation” often refers to the process of investigating the conceptual equivalence and content validity of translation of PRO instruments, including forward- and back-translation, review, harmonization, and cognitive debriefing [19, 20]; it does not include psychometric validation, which is nonetheless seen as an important second step for cross-cultural validation [21]. This linguistic validation study is part of a cross-cultural validation; psychometric evaluation was conducted in another study with a different 180 participants (UMIN CTR: UMIN000015169). The results of psychometric validation are beyond the scope of the current report and will be presented separately.

## Methods

### Translation procedure

The PRO-CTCAE questionnaire was translated into Japanese following the guidance of the International Society for Pharmacoeconomics and Outcomes Research (ISPOR) on the “Translation and Cultural Adaptation Process for PRO Measures” [20]. Two forward-translations were independently produced by two native-Japanese-speaking language professionals, after which a single reconciled version was created by the project manager and these two translators. Two native English-speakers who are fluent in Japanese then produced independent back-translations. An independent review was performed by the JCOG Executive Committee, and the finalized Japanese translation was reviewed and approved by a bilingual medical oncologist at the US NCI.

### Participants and setting

Patients were eligible for the study if they were 18 years of age or older; had been diagnosed with cancer; were receiving or had received chemotherapy, radiotherapy, or hormone therapy within the past 6 months at one of the study sites; and were able to read, write, and answer survey items and interview questions verbally in Japanese as their first language.

Patients were excluded if the physician determined that they had cognitive impairments or were incapable of participating in the study. Patients were recruited at the Kansai Medical University Hirakata Hospital and The University of Tokyo Hospital. Regarding the sample size determination, as a literature review of guidelines suggested that the required sample size of cognitive interviews for translation of HQOL questionnaire is around 5 to 15 [19], we aimed to include more than 16 patients while reflecting differences in cancer type, gender, regional characteristics, and educational background.

### Cognitive interview goal and procedure

In total, 123 translated items were divided into two questionnaires (A and B) per symptom, to reduce participant burden. Although the PRO-CTCAE library consists of 124 items, the item “vaginal dryness” was not included in the cognitive interviews as it was a sexually sensitive question. As six items are gender-specific, two versions of each questionnaire were prepared. Furthermore, six symptoms (nine items) were shifted from questionnaire A to B when the cognitive interviews took place at the University of Tokyo Hospital to minimize the item number imbalance between questionnaires; a total of eight questionnaires were prepared for the cognitive interviews. Each questionnaire consists of about 61 items on average (50 items at minimum and 68 at maximum).

The cognitive interviews were conducted by master’s-level psychologists with field experience. The following study procedures were followed: 1) investigators at the site screened patients based on the eligibility criteria; 2) interviewers explained the details of the study to participants and obtained informed consent; 3) interviewers scheduled interviews; 4) on the day of the interview, interviewers reconfirmed participants’ willingness to participate; 5) participants were asked to fill out a gender-specific questionnaire; and 6) one-on-one semi-structured interviews were conducted.

Participants were asked to self-complete the PRO-CTCAE and make remarks on the items and response options they evaluated as “difficult to understand” or “difficult to answer” regarding: 1) comprehensibility; 2) clarity; 3) knowledge and recall (ease of memory retrieval); and 4) judgment (adequacy of response options). Participants were then interviewed using semi-structured scripts and predetermined probes to investigate any items that were difficult to comprehend if the response choices and recall period were clear and relevant to their experiences. Reluctance or hesitation to answer and spontaneous questions, responses, or facial expressions while answering were also observed by the interviewer. The interviews took place in a private room.

### Analytical approach

We conducted a qualitative-descriptive analysis using semi-structured cognitive interviews. The interviews were transcribed word-for-word, examined repeatedly, and categorized. Remarks were split into two categories: 1) remarks on the items and 2) remarks on the questionnaire in general. The remarks on the items were further categorized into those concerning 1) comprehension difficulties, 2) clarity, 3) knowledge and recall (ease of memory retrieval), and 4) ease of judgment and other spontaneous comments. The remarks on the questionnaire were also further categorized into those concerning 1) attributes, 2) ease of memory retrieval and judgment, 3) other remarks on the questionnaire, and 4) other remarks in general. As a single patient could make several remarks, the total number of remarks does not reflect the number of patients. We performed descriptive statistics to summarize the variables for the cognitive interviews.

## Results

### Translation procedure

Comparing the two independent forward-translations, 52 of 80 PRO-CTCAE symptom terms were fully agreed upon or conceptually equivalent. In the reconciliation process, the term “how often” in the frequency attribute items was altered so as not to be directly expressed, due to an element of the Japanese cultural background. In Japanese custom, asking about frequency of symptoms before asking about the presence of symptoms, that is, assuming their presence, is unnatural and impolite. The use of “how often” was flagged by the JCOG executive committee review, and so in the further revision process, we agreed to not translate “how often” directly in the question but instead to refer to “frequency” in the response options. For severity attribute items, translation of “its worst” became a discussion topic during the forward-translation reconciliation process. In Japanese, there are objective and subjective terms corresponding to the term “its worst”; for the test script, we changed “its worst” to “*hidoi*,” which is an objective term, and further solicited opinions on two options in the cognitive interviews. In the review by the bilingual medical oncologist from NCI, there were no major suggestions for change. One minor suggestion was made to add supplementary explanation to the “radiation skin reaction” item indicating that this question is intended for cancer patients receiving radiation therapy.

### Cognitive interviews

A total of 21 cancer patients (six males and 15 females) participated in interviews at either the University of Tokyo Hospital or the Kansai Medical University Hirakata Hospital between August 2011 and April 2012. Table 1 shows the clinical and demographic characteristics of the participants. The patients were 40 to 80 years old, with a mean of 64 years. Ten had been diagnosed with breast cancer, seven with lung cancer, three with pancreatic cancer, and one with esophageal cancer. All participants except one, who was receiving hormone therapy, were undergoing chemotherapy at the time of the interview. Nineteen participants had been receiving the cancer treatment less than 1 month, one participant for 1 to 3 months, and one participant for more than 12 months. The Eastern Cooperative Oncology Group (ECOG) performance status was 0 for 9 participants, 1 for 8 participants, 2 for 2 participants, and 3 for 2 participants. The participants took an average of 30 min to complete the questionnaires of approximately 61 items; skip patterns were not employed.

**Table 1:** Patient characteristics

Characteristics	*N* = 21 (%)
Gender (n, %)	
Male	6 (29%)
Female	15 (71%)
Age in years, mean (SD, range)	64 (11.2, 40–80)
Recruitment site (n, %)	
The University of Tokyo Hospital	11 (52%)
Kansai Medical University Hospital	10 (48%)
Primary lesion (n, %)	
Breast	10 (48%)
Lung	7 (33%)
Pancreatic	3 (14%)
Esophageal	1 (5%)
Progression (n, %)	
Localized	3 (14%)
Metastatic	18 (86%)
ECOG Performance Status (n, %)	
Grade 0	9 (43%)
Grade 1	8 (38%)
Grade 2	2 (10%)
Grade 3	2 (10%)
Treatment modality (n, %)	
Chemotherapy	20 (95%)
Hormone therapy	1 (5%)
Treatment time (n, %)	
within 1 month	19 (90%)
1–3 months prior	1 (5%)
12 months prior	1 (5%)
Employment (n, %)	
Employed	3 (14%)
Housewife	10 (48%)
Unemployed	6 (29%)
Others	2 (10%)
Education	
Junior high school	7 (33%)
High school	5 (24%)
Junior college	6 (29%)
Undergraduate/postgraduate	3 (14%)
Questionnaire^a^ completion time in minutes, mean (SD, range)	30 (11.5, 15–58)

^a^61 items on average (minimum 50, maximum 68)

Table 2 lists the questionnaire items, showing the proportion of patients who evaluated each PRO-CTCAE item as “difficult to understand” and “difficult to answer” respectively. The patients self-completed 45 items without any difficulties; 33 items were evaluated as “difficult to understand,” and 65 as “difficult to answer.” Only one PRO-CTCAE item (anxiety severity) was found to be “difficult to understand” by more than 20% of the respondents. Eight items were evaluated as “difficult to answer” by more of 20% of the respondents; three of these were items related to sexual activities, such as “decreased sexual interest,” “unable to have an orgasm or climax,” and “took too long to have an orgasm or climax.”

**Table 2:** Proportion of patients who evaluated items as “difficult to understand” or “difficult to answer”

Items	Dimension	Difficult to understand		Difficult to answer	
		n/N	%	n/N	%
ACHING JOINTS	Frequency	0/10		0/10	
ACHING JOINTS	Severity	0/10		1/10	10.0%
ACHING JOINTS	Interference	0/10		1/10	10.0%
ACHING MUSCLES	Frequency	0/10		0/10	
ACHING MUSCLES	Severity	0/10		1/10	10.0%
ACHING MUSCLES	Interference	0/10		1/10	10.0%
ACNE OR PIMPLES ON THE FACE OR CHEST	Severity	2/10	20.0%	1/10	10.0%
ANXIETY	Frequency	1/11	9.1%	1/11	9.1%
ANXIETY	Severity	3/11	27.3%	3/11	27.3%
ANXIETY	Interference	1/11	9.1%	2/11	18.2%
ARM OR LEG SWELLING	Frequency	0/16		1/16	6.3%
ARM OR LEG SWELLING	Severity	0/11		2/11	18.2%
ARM OR LEG SWELLING	Interference	0/11		2/11	18.2%
BED SORES	Presence	0/11		0/11	
BLOATING OF THE ABDOMEN (BELLY)	Frequency	0/10		0/10	
BLOATING OF THE ABDOMEN (BELLY)	Severity	0/10		1/10	10.0%
BLURRY VISION	Severity	0/10		3/10	30.0%
BLURRY VISION	Interference	0/10		0/10	
BODY ODOR	Severity	1/11	9.1%	3/11	27.3%
BREAST AREA ENLARGEMENT OR TENDERNESS	Severity	0/10		0/10	
BRUISE EASILY (BLACK AND BLUE MARKS)	Presence	0/10		2/10	20.0%
CHANGE IN THE COLOR OFYOUR FINGERNAILS OR TOENAILS	Presence	0/11		0/11	
CONSTIPATION	Severity	0/11		1/11	9.1%
COUGH	Severity	0/11		0/11	
COUGH	Interference	0/11		1/11	9.1%
DECREASED APPETITE	Severity	0/11		0/11	
DECREASED APPETITE	Interference	1/11	9.1%	3/11	27.3%
DECREASED SEXUAL INTEREST	Severity	0/10		5/10	50.0%
DIFFICULTY GETTING OR KEEPING AN ERECTION	Severity	0/6		0/6	
DIFFICULTY SWALLOWING	Severity	0/11		1/11	9.1%
DIZZINESS	Severity	0/11		0/11	
DIZZINESS	Interference	0/11		1/11	9.1%
DRY MOUTH	Severity	0/11		0/11	
DRY SKIN	Severity	0/10		1/10	10.0%
EJACULATION PROBLEMS	Presence	1/6	16.7%	0/6	
FATIGUE, TIREDNESS, OR LACK OF ENERGY	Severity	0/11		0/11	
FATIGUE, TIREDNESS, OR LACK OF ENERGY	Interference	1/11	9.1%	1/11	9.1%
FEEL THAT NOTHING COULD CHEER YOU UP	Frequency	0/11		1/11	9.1%
FEEL THAT NOTHING COULD CHEER YOU UP	Severity	1/11	9.1%	1/11	9.1%
FEEL THAT NOTHING COULD CHEER YOU UP	Interference	1/11	9.1%	1/11	9.1%
FLASHING LIGHTS IN FRONT OF YOUR EYES	Presence	0/10		0/10	
FREQUENT URINATION	Frequency	0/11		0/11	
FREQUENT URINATION	Interference	0/11		1/11	9.1%
HAIR LOSS	Amount	1/11	9.1%	1/11	9.1%
HAND-FOOT SYNDROME	Severity	0/11		0/11	
HEADACHE	Frequency	0/11		0/11	
HEADACHE	Severity	0/11		1/11	9.1%
HEADACHE	Interference	0/11		1/11	9.1%
HEARTBURN	Frequency	0/10		0/10	
HEARTBURN	Severity	0/10		1/10	10.0%
HICCUPS	Frequency	0/11		0/11	
HICCUPS	Severity	0/11		1/11	9.1%
HIVES (ITCHY RED BUMPS ON THE SKIN)	Presence	1/10	10.0%	0/10	
HOARSE VOICE	Severity	1/11	9.1%	0/11	
HOT FLASHES	Frequency	1/10	10.0%	1/10	10.0%
HOT FLASHES	Severity	1/10	10.0%	1/10	10.0%
INCREASED PASSING OF GAS (FLATULENCE)	Presence	0/10		1/10	10.0%
INCREASED SKIN SENSITIVITY TO SUNLIGHT	Presence	1/10	10.0%	0/10	
INSOMNIA	Severity	0/11		1/11	9.1%
INSOMNIA	Interference	0/11		0/11	
MISS AN EXPECTED MENSTRUAL PERIOD	Presence	0/5		1/5	20.0%
IRREGULAR MENSTRUAL PERIODS	Presence	0/5		0/5	
ITCHY SKIN	Severity	1/11	9.1%	0/11	
LOOSE OR WATERY STOOLS (DIARRHEA)	Frequency	0/11		1/11	9.1%
LOSE ANY FINGERNAILS OR TOENAILS	Presence	1/10	10.0%	1/10	10.0%
LOSS OF CONTROL OF BOWEL MOVEMENTS	Frequency	0/10		0/10	
LOSS OF CONTROL OF BOWEL MOVEMENTS	Interference	0/10		0/10	
LOSS OF CONTROL OF URINE (LEAKAGE)	Frequency	0/11		1/11	9.1%
LOSS OF CONTROL OF URINE (LEAKAGE)	Interference	0/11		1/11	9.1%
MOUTH OR THROAT SORES	Severity	0/11		0/11	
MOUTH OR THROAT SORES	Interference	0/11		1/11	9.1%
NAUSEA	Frequency	0/11		0/11	
NAUSEA	Severity	0/11		1/11	9.1%
NOSEBLEEDS	Frequency	0/10		0/10	
NOSEBLEEDS	Severity	0/10		0/10	
NUMBNESS OR TINGLING IN YOUR HANDS OR FEET	Severity	1/11	9.1%	1/11	9.1%
NUMBNESS OR TINGLING IN YOUR HANDS OR FEET	Interference	1/11	9.1%	0/11	
PAIN	Frequency	0/11		1/11	9.1%
PAIN	Severity	0/11		2/11	18.2%
PAIN	Interference	0/11		2/11	18.2%
PAIN DURING VAGINAL SEX	Severity	0/5		0/5	
PAIN IN THE ABDOMEN (BELLY AREA)	Frequency	0/11		0/11	
PAIN IN THE ABDOMEN (BELLY AREA)	Severity	0/11		1/11	9.1%
PAIN IN THE ABDOMEN (BELLY AREA)	Interference	0/11		1/11	9.1%
PAIN OR BURNING WITH URINATION	Severity	0/10		0/10	
PAIN, SWELLING, OR REDNESSAT A SITE OF DRUG INJECTION OR IV	Presence	0/11		0/11	
POUNDING OR RACING HEARTBEAT (PALPITATIONS)	Frequency	0/10		1/10	10.0%
POUNDING OR RACING HEARTBEAT (PALPITATIONS)	Severity	0/10		2/10	20.0%
PROBLEMS WITH CONCENTRATION	Severity	1/11	9.1%	1/11	9.1%
PROBLEMS WITH CONCENTRATION	Interference	1/11	9.1%	1/11	9.1%
PROBLEMS WITH MEMORY	Severity	2/10	20.0%	1/10	10.0%
PROBLEMS WITH MEMORY	Interference	1/10	10.0%	0/10	
PROBLEMS WITH TASTING FOOD OR DRINK	Severity	0/11		1/11	9.1%
RASH	Presence	0/11		0/11	
RIDGES OR BUMPS ON YOUR FINGERNAILSOR TOENAILS	Presence	0/10		2/10	20.0%
RINGING IN YOUR EARS	Severity	0/10		0/10	
SAD OR UNHAPPY FEELINGS	Frequency	0/11		1/11	9.1%
SAD OR UNHAPPY FEELINGS	Severity	1/11	9.1%	2/11	18.2%
SAD OR UNHAPPY FEELINGS	Interference	0/11		1/11	9.1%
SHIVERING OR SHAKING CHILLS	Frequency	0/10		1/10	10.0%
SHIVERING OR SHAKING CHILLS	Severity	0/10		0/10	
SHORTNESS OF BREATH	Severity	1/10	10.0%	0/10	
SHORTNESS OF BREATH	Interference	1/10	10.0%	0/10	
SKIN BURNS FROM RADIATION	Severity	0/10		4/10	40.0%
SKIN CRACKING AT THE CORNERS OF YOUR MOUTH	Severity	1/10	10.0%	0/10	
SPOTS OR LINES THAT DRIFT IN FRONT OF YOUR EYES (FLOATERS)	Presence	0/10		0/10	
STRETCH MARKS	Presence	2/10	20.0%	0/10	
SUDDEN URGES TO URINATE	Frequency	1/10	10.0%	0/10	
SUDDEN URGES TO URINATE	Interference	1/10	10.0%	0/10	
TOOK TOO LONG TO HAVE AN ORGASM OR CLIMAX	Presence	0/10		4/10	40.0%
UNABLE TO HAVE AN ORGASM OR CLIMAX	Presence	0/10		4/10	40.0%
UNEXPECTED DECREASE IN SWEATING	Presence	1/10	10.0%	1/10	10.0%
UNEXPECTED OR EXCESSIVE SWEATINGDURING THE DAY OR NIGHTTIME	Frequency	0/10		0/10	
UNEXPECTED OR EXCESSIVE SWEATINGDURING THE DAY OR NIGHTTIME	Severity	0/10		0/10	
UNUSUAL DARKENING OF THE SKIN	Presence	0/10		0/10	
UNUSUAL VAGINAL DISCHARGE	Presence	0/5		0/5	
URINE COLOR CHANGE	Presence	0/10		0/10	
VOICE CHANGES	Presence	0/10		0/10	
VOMITING	Frequency	0/10		0/10	
VOMITING	Severity	0/10		0/10	
WATERY EYES (TEARING)	Severity	1/10	10.0%	1/10	10.0%
WATERY EYES (TEARING)	Interference	0/10		0/10	
WHEEZING	Severity	0/10		0/10	

Analyzing the 94 narrative comments provided on the items across the debriefing interviews, 11 remarks concerned comprehension difficulties, 13 remarks concerned clarity, 25 remarks concerned knowledge and recall (ease of memory retrieval), and 45 remarks concerned ease of judgment or spontaneous comments. These remarks thus applied to 52 out of the 123 items, with 71 items not remarked on. Patients found it difficult to interpret some symptoms, such as “body odor” (5 remarks) or “problems with memory” (2 remarks), because their effects are relatively subjective. Determining the severity of persistent symptoms such as “anxiety” (4 remarks) or “leg/arm swelling” (1 remark) was also problematic for small numbers of participants. Problems determining whether something like “aching joints” included symptoms such as stiff shoulders or aching muscles were reported by five patients.

The following remarks related to the symptom attribute (40 remarks) in the questionnaire were categorized: no evaluation criteria are given (5 remarks), need for benchmark (6 remarks), difficulty choosing only one response (4 remarks), distinction between response options is ambiguous (7 remarks), response options are inadequate (6 remarks), questions and response options need to be reformed (7 remarks), and inconsistency between questions and response options (4 remarks), and other (1 remarks). The following remarks related to ease of memory retrieval and judgment (26 remarks) were categorized: some symptoms are hard to notice (6 remarks), need for exemplars of symptoms (10 remarks), significance of the question should be clarified (3 remarks), problems interpreting “interference with daily activities” (4 remarks), and problems apprehending the severity of symptoms at their worst (3 remarks). Other remarks on the questionnaire include the phrase “in the last 7 days,” which complicated questions (5 remarks). Participants with constant subjective symptoms in particular seemed to find it challenging to confine the interpretation of their symptom experience to only the severity or interference experienced “in the last 7 days.” Regarding the two alternative wordings of “its worst,” the responses were varied among responders and symptoms. Following agreement on changes among the project team, ultimately we kept the initial wording, which is the objective term discussed above, in the final version.

## Discussion

This study was conducted following rigorous procedures for translation and linguistic validation [20]. Although the methodologies used were consistent with other translations of PRO-CTCAE, our sample was relatively small compared to those of other linguistic validation studies [13–15]. However, the study population partially represents the clinical, socioeconomic, and geographic diversities in the general Japanese cancer population. Moreover, there were no missing data, and the feasibility of the measurements was confirmed.

In total, 33 items were evaluated as “difficult to understand” and 65 items were evaluated as “difficult to answer” by one or more participants when the participants completed the questionnaire independently. However, when we asked them about these items in the interviews, only 24 out of their 94 remarks were categorized as relating to “comprehension difficulties” or issues with “clarity.” For the qualitative analysis, we set the threshold for consideration of rephrasing and testing of alternative phrasing at more than 20%. Table 3 shows the remarks on the nine PRO-CTCAE items that were evaluated as “difficult to understand” or “difficult to answer” by more than 20% of the respondents. Although some remarks on “anxiety” were categorized as “clarity,” they were related to the apprehension of symptomatic toxicities in the context of attributes, such as “the severity of anxiety at its worst.” Anxiety items were also found to be challenging in Spanish PRO-CTCAE cognitive testing [13]. Remarks on other items were mostly related to problems with interpreting the symptom itself and judging individual symptomatic events. Nevertheless, we concluded that there was a good general understanding of these items, so no amendments to the Japanese translated version were required after the cognitive interviews.

**Table 3:** PRO-CTCAE items evaluated as “difficult to understand” or “difficult to answer” by more than 20% of patients and their remarks

Item	Attribute	Patients with difficulty (%)	No. of remarks coded^a^	Comprehension	Clarity	Knowledge/recall	Ease of judgment	Examples of patient remarks
Evaluated as “difficult to understand” by >20% of patients								
Anxiety	Severity	3/11 (27%)	6	–	2	3	2	Cannot apprehend severity of worst anxiety
Evaluated as “difficult to answer” by >20% of patients								
Anxiety	Severity	3/11 (27%)	6	–	2	3	2	To know my anxiety at its worst is difficult due to ambiguous and constant nature of symptom
Blurry vision^b^	Severity	3/10 (30%)	0	.	.	.	.	–
Body odor	Severity	3/11 (27%)	5	.	.	5	.	I am not sure if I should give my own evaluation or third-person’s evaluation
Decreased appetite	Interference	3/11 (27%)	2	–	.	2	.	Hard to imagine a situation where decreased appetite interferes with daily activities
Decreased sexual interest	Severity	5/10 (50%)	4	.	.	1	3	Unsure if decreased sexual interest resulting from the aging process should be included
Skin burns from radiation^b^	Severity	4/10 (40%)	0	.	.	.	.	–
Unable to orgasm/climax	Presence	4/10 (40%)	1	.	.	.	1	This is a question for patients on lower stages, not for those on 4th stage
Took too long to climax^b^	Presence	4/10 (40%)	0	.	.	.	.	–

^a^Multiple remarks could be coded from a single patient

^b^Several items elicited no remarks in interviews even though they were evaluated as difficult to understand or answer in the initial investigation

Items related to sexual activities, such as “decreased sexual interest,” “unable to have an orgasm or climax,” and “took too long to have an orgasm or climax,” were evaluated as “difficult to answer” by more than 40% of patients. Moreover, “unable to have an orgasm or climax” was remarked on only once, and “took too long to have an orgasm or climax” was not remarked on at all, even though a high proportion of patients evaluated them as “difficult to answer.” This may have been because Japanese patients tend to be hesitant about answering questions regarding sexual activities [22]. The lack of remarks on these items could also have been influenced by gender non-concordance between study participants and interviewers.

As discussed above, the patients comprehended the questions and response items well in general. However, interpreting symptoms using given response options was confusing for some. For example, three patients had issues with the severity-related wording “at its worst.” Chronic symptoms seemed to be especially difficult to evaluate in this regard. There were opinions that the inclusion of “mild” as a response option was not consistent with asking about the severity of a symptom “at its worst.” Similar finding with severity items were reported in cognitive interviews in an adolescent population in USA [11]. Considering remarks on the questionnaire such as “need for a benchmark” or “distinction among response options is ambiguous,” it is clear that some patients find it difficult to respond using abstract terminology instead of clear benchmarks, which may reflect the sociocultural matter of Japanese respondents’ limited experience with patient-reported outcome measurements in general rather than the Japanese translation. Cultural differences exist in how Likert-type scales are responded to, and Japanese people have frequently reported difficultly doing so [23]. Also, 4 out of 6 remarks on “need for a benchmark” were related to psychological symptoms, including sleep, mood and memory, and sensory aspects, and patients might have felt more difficulty choosing options for these compared to other types of symptoms. Nevertheless, the cognitive interviews showed a good general understanding of most of the items.

These study results should be interpreted with several caveats in mind, including the small overall sample size, the small number of respondents interviewed about each item, and the possibility that the use of gender-discordant interviewers may have made some participants reticent to discuss difficulties with PRO-CTCAE items that reflect sexual functioning and genitourinary symptoms. Also, we could not include Japanese-speaking cancer patients living in the USA, who were part of the target population of this questionnaire in the study. These caveats notwithstanding, this study provides preliminary evidence supporting the linguistic validity of the Japanese language version of PRO-CTCAE. These results should be confirmed and elaborated on with further psychometric validation studies covering construct validity, test–retest reliability, and sensitivity to detect change; such studies are currently in progress as a part of the larger cross-cultural validation of which this study is a part. The translation certificate was issued by the NCI, and the first version of the Japanese translation is currently available through the PRO-CTCAE website [7].

## Conclusions

This study revealed that translation was conducted using a rigorous process and the majority of the items in the Japanese language version of PRO-CTCAE are well understood by Japanese-speaking cancer patients. The results provide preliminary evidence supporting the linguistic and content validity of the Japanese language version of PRO-CTCAE. However, further cognitive interviews are needed for items related to sexuality and to anxiety severity, as well as response options for severity attribute items.
